# Old-Fashioned Pauper Infirmaries

**Published:** 1886-11-06

**Authors:** 


### Old-fashioned Pauper Infirmaries.

Better Sentiments.

Religion, some nervous souls believe, is dying out of the land. That we are getting less dogmatic and pugnacious in our religion is, no doubt, true. But on all hands there are signs and evidences that while we are growing less and less inclined to roast each other for differences in the rendering of Greek texts, and less and less cocksure about our individual solutions of some of the profoundest mysteries of the universe, we are also growing more humane and sympathetic; more sensitive to the promptings of pity and compassion; more considerate of the poor and helpless; more alive to their needless sufferings, and more grimly impatient of the hardness and injustice of their lot. That phase of religion, at any rate, is breaking brighter and brighter through the darkness; its lustre is daily spreading wider and wider, and under its shining light, cruelty, inhumanity, and oppression are visibly dwindling away.

Twenty Years Ago.

I was very forcibly impressed with a conviction of this as I stood the other day in one of the wards of the splendid infirmary which was opened in 1881 for the benefit of the sick poor of the parish of St. Marylebone, and thought of some of the horrors characteristic of the old system of things. It is barely more than twenty years ago since it was said, with perfect truth, that in dietary, in nursing, in medical supervision, in general management, in fittings and appointments, workhouse hospitals, even the best of them, were scandalously defective, while the pertinacity and unanimity with which Bumbledom in all its grades refused to listen to all appeals for greater humanity and care of the sick poor were with equal truth declared to be the most damning characteristic of the internal administration of this country. Many of the features of workhouse infirmary management, even in the heart of London, were almost too revolting and offensive to be presented to the general reader. There was then hardly an establishment of the kind in London in which classification and separation, and the ventilation of wards, received any real attention. In many of them typhus and small-pox patients were admitted indiscriminately with others, and in some instances this scandalously cruel and stupid negligence was aggravated by overcrowding. It is a curious illustration of the old-fashioned estimate of the pauper—

"Rattle his bones over the stones,
He's only a pauper whom nobody owns"—

that even the Poor-law, theoretically so studious of everything affecting life and health, should deliberately have sanctioned the hustling together of the sick poor into spaces known to be insufficient for them. A cubic space of 1,000 feet is insisted on as the minimum for each patient in our great hospitals, and in the public voluntary hospitals of London the space allotted ranges from 1,000 to 1,300 cubic feet. For the unfortunate pauper 500 feet was taken to be enough—a space about 10 feet long, 10 feet high, and 5 feet wide—and, as a matter of course, under the management of Poor-law Guardians, these space-limits often closed in, like the iron walls of the Inquisition, upon the hapless patient struggling for life and health in an atmosphere heavy with the germs of disease and thick with the products of respiration. In St. Martin's-in-the-Fields, before the old workhouse was abandoned, they got the average down to 428 feet per patient, and that, too, in the surgical wards, situated in the basement beneath the level of a disused burial-ground. Some London guardians were not content with poisoning and asphyxiating their patients by huddling them together nearly three times as close as they should have been. They contrived to add a touch of torture by external means. At the Strand Workhouse, for instance, in a miserably unsatisfactory building, there were, ordinarily, some 200 people seriously ill and 400

chronically infirm and ailing, and underneath the windows of the wards in which these poor mortals, with their shattered nerves and aching heads, were in bed, the guardians actually set up a large carpet-beating business. "Culpable thoughtlessness!" exclaims the reader. Not a bit of it. Again and again the inhuman disregard of the comfort of the poor patients was charged against the guardians, and they were entreated to abate a nuisance which tormented the sick and feeble and dying with noise, and choked them with dust, or compelled them to keep all their windows closed. But the carpet-beating was a matter of six hundred a year to these precious "guardians" of the poor, and they kept it up for years.

An inadequate Medical Staff.

One source of incalculable mischief in the management of these places at the time we are speaking of —and we do not for a moment wish to be understood to imply that it is a source entirely removed even now—was the inadequacy of the medical staff, and the wretched parsimony displayed in the payment of medical services. In only about one-fourth of the London workhouses was there a resident medical officer, and in the great majority of them medical attendance was little more than a ghastly farce. For instance, in one large establishment, with an average of 300 cases of acute sickness, and 600 patients, more or less infirm, and in need of medical care, the entire staff consisted of one surgeon and one assistant, and they were paid to attend the hospital only in the intervals of private practice. At Greenwich it was said that there were 900 aged and infirm people, and 400 actually ill, and only one non-resident doctor to attend to them all, in the time he could spare from his private practice. At Shoreditch there were 700 infirm, over 200 sick, and 140 insane, epileptic, and imbecile, and they were all supposed to be under the care of one man, who came for a couple of hours in the morning. Of course the poor man had no time to write prescription cards; he had to trust his memory from day to day, and such directions as he could find time to give were supposed to be remembered and carried out by pauper nurses—many of them paupers simply because they were idle and vicious, and, as it was shown in connection with another workhouse, in nine cases out of eleven, given to drunkenness.

The Pauper Nurses.

What grim jokes a De Quincey would have cracked over the blunders, brutalities, and the peculations of these pauper nurses! Think of the helpless invalids who would be popped into scalding baths; of the patients who should have had quinine and got ipecacuanha; of moaning sufferers who were tortured day after day by blear-eyed "nurses," who owed them a grudge, and who fuddled on the stimulants their victims were dying for want of. Twenty years ago one of the male nurses justified himself thus, for allowing a poor wretch, who had tumbled out of bed in a fit, to die on the floor without assistance. "He were dead enough, he were," said the nurse, "and wot were the use o' rousing Mr. Blunt or anybody else? *They* couldn't bring a dead man to life again; not they. Why should they be disturbed? 'Ow did I know he were dead? Why, I shook 'im and he never answered, that's how. Was the body cold when I saw it? Yes, it were; leastwise the feet was quite cold." All this, said the witness, with a smile assumptive of wisdom, judgment, and tact inexpressibly given.

No Night Nurses.

Twenty years ago, not only were most of the sick paupers in London left during the day pretty much to the tender mercies of pauper inmates, who were always ignorant and untrained, and usually more or less embittered and vicious, and whose emoluments were extra food and a liberal allowance of beer and gin; but it appears to have been a fact that at least in some of the workhouse infirmaries there was by night no attendance at all. Imagine from three hundred to four hundred people, with almost every form and phase of human malady among them, and never a soul near them through the weary hours of night to moisten a lip, or ease a posture, or give the slightest help! One entirely credible witness says that in one ward he found two paralytic patients with fearful sloughs on the back. They were both dirty, and lying on hard straw mattresses; one dressed only with a rag steeped in chloride of zinc solution, the other with a rag thickly covered with ointment. This latter was a fearful and very extensive sore in a state of absolute putridity; and other details were given, which may be sufficiently gathered from the fact that to mask the stench arising from the bed chloride of lime was strewed under it. Hideous and revolting as this must be to every humane sentiment, it affords by no means an exaggerated idea of the state of things often to be met with in pauper infirmaries in the heart of this Christian land of ours only twenty years ago.

To-day.

Now there are some forty or fifty workhouses in London, and it would be very rash indeed, even for a person who has some acquaintance with them all, to pronounce positively as to what does or does not go on within some of their infirmaries. But it is not too much to say that the whole tone of feeling has, during the past few years—thanks, in a very great measure, to the humane and persistent efforts of the medical profession—undergone a great change, as will be shown in another article.

---

Dentistry.—"How do you get your living?" was the question asked the other day in a police-court. "I live from hand to mouth." "What sort of a trade do you call that?" "Why, dentistry, of course!"

